# Use of Intravitreal Methotrexate Infusion in Complicated Retinal Detachment for Prevention of Proliferative Vitreoretinopathy in a Pilot Study

**DOI:** 10.7759/cureus.17439

**Published:** 2021-08-25

**Authors:** Sana Jahangir, Tehmina Jahangir, Muhammad H Ali, Qasim Lateef, Uzma Hamza, Haroon Tayyab

**Affiliations:** 1 Ophthalmology, Vitreoretinal Surgery, Allama Iqbal Medical College/Jinnah Hospital, Lahore, PAK; 2 Ophthalmology, Vitreoretinal Surgery, Lahore General Hospital, Lahore, PAK; 3 Surgery, Ophthalmology, The Aga Khan University Hospital, Karachi, PAK

**Keywords:** retina, methotrexate, proliferative vitreoretinopathy, retinal detachment, silicone oil, retinal pigment epithelium, tractional retinal detachment

## Abstract

Objective

The objective of this pilot study was to evaluate the efficacy and safety of per-operative intravitreal methotrexate (MTX) infusion during vitrectomy in patients of retinal detachment (RD) with advanced grade proliferative vitreoretinopathy (PVR).

Methods

In this prospective interventional case series, we included patients with Grade C PVR, recurrent RD, and open globe trauma. All patients underwent standard single surgeon operated 23-gauge pars plana vitrectomy (PPV) with 80mg of MTX in 1000mL of irrigation fluid. All patients were followed up after four months to assess the final status of retinal attachment and visual acuity. Ethical review board permission was sought for this off-label use of MTX and all patients signed an informed consent form before this intervention.

Results

Thirty eyes of 30 patients with recurrent retinal detachment, open globe trauma, or grade C PVR at initial presentation were included in this study. After PPV, these patients were followed up after four months. A total of 24 (80%) patients maintained retinal attachment at four months. Mean preoperative best-corrected visual acuity (BCVA) was 1.35 logarithm of the minimum angle of resolution (logMAR) (range 0.5-3) and mean four months postoperative BCVA was 1.01 logMAR (range 0.3-3) (Student’s *t*-test; *P*-value <0.05). Seventeen (56.6%) eyes had pre-operative BCVA of 1.0 whereas 25 (83.3%) had BCVA of 1.0 at the end of the follow-up period. Six (20%) patients had preoperative BCVA of 0.7 whereas 12 (40%) patients had BCVA of 0.7 at four months postoperatively. Out of six (20%) eyes developing RD after this intervention, four eyes achieved retinal reattachment after a second surgery. We did not observe any MTX-related complications during the follow-up period of this study.

Conclusion

Intravitreal MTX infusion during PPV for complicated RD as an adjunctive therapy showed encouraging results and was found to be safe in its use. We need more rigorous and controlled studies to confirm the possible advantages of MTX and its role in the prevention of PVR.

## Introduction

The leading cause of recurrent retinal detachment (RD) is proliferative vitreoretinopathy (PVR). It is associated with the progression of fibrosis and contraction of fibrous membranes. The growth of PVR membranes is driven by the retinal pigment epithelial (RPE) cells, pro-inflammatory and retinal glial cells and these membranes grow on both surfaces of the retina, within the retina, and in the vitreous cavity [1]. These membranes tend to contract and thus lead to the formation of new retinal breaks and recurrent retinal detachment. Also, these membranes prevent initial retinal reattachment as well [2]. PVR is present in five to 10% of all retinal detachments and it affects 75% of all retinal detachments appearing within one month of RD surgery [3,4]. The structural success rate of retinal reattachment surgery is reported to be between 45 to 85% after PVR-associated retinal detachment repair and the functional success is limited to 26 to 67% [5,6].

There are a lot of risk factors that have been associated with accelerated PVR. The most common preoperative risk factors include intraocular inflammation, low intraocular pressure, large retinal breaks, multiple retinal breaks, the extensive extent of retinal detachment, and preexisting PVR. Intraoperative risk factors include RPE dispersion during surgery, subretinal hemorrhage, and excessive retinopexy using cryotherapy or laser. Postoperative risk factors include choroidal detachment, use of sulfur hexafluoride, prolonged inflammation, and multiple retinal surgeries [7-9].

We currently follow the PVR grading scheme proposed by The Retina Society Terminology Committee in 1991. It divides PVR into grades A, B, and C. The main cascade of PVR initiates from the breakdown of the blood-retinal barrier (BRB) and retinal hypoxia. RPE cells migrate to the retinal surface through the break and initiate their interaction with migrating retinal glial cells [10]. The breakdown of BRB leads to complex interactions of chemotactic and mitogenic factors with PRE and glial cells. Proinflammatory cells involved in PVR formation include T and B lymphocytes, macrophages, and major histocompatibility cells. Certain growth factors also enter the vitreous cavity through impaired BRB. These include platelet-derived growth factor (PDGF), hepatocyte growth factor (HGF), vascular endothelial growth factor (VEGF), epidermal growth factor (EGF), granulocyte-colony stimulating factor (G-CSF), acidic and basic fibroblastic growth factor (aFGF and bFGF), insulin-like growth factor 1 (IGF-1), connective tissue growth factor (CTGF), transforming growth factor α (TGF-α), transforming growth factor β (TGF-β), tumor necrosis factor α (TNF-α), interferon β (IFN-β), interferon γ (IFNγ) and others [11].

Currently, there is no proven treatment of PVR or for its prevention. Corticosteroids in topical, intravitreal and sustained-release (Ozurdex, Allergan Inc, Irvine, CA) form have been tested in various clinical trials but their efficacy is still not proven [12]. Various antineoplastic and antiproliferative agents have been tested for the prevention and treatment of PVR. These include daunorubicin, colchicine, vincristine, adriamycin, 5-fluorouracil (5-FU), mitomycin, and cisplatin. Out of these, 5-FU has been one of the most extensively studied compounds. But it has also failed to show conclusive evidence of its role in preventing PVR. In fact, it was associated with various side effects in human studies [13,14].

Similarly, work has been done to evaluate the ability of anti-vascular endothelial growth factors (anti-VEGF) to prevent PVR. Experiments with Bevacizumab have shown no superiority in functional outcomes, re-detachment rates, and PVR formation between trial and control groups across various grades of PVR [15,16].

Recently, methotrexate (MTX) has been extensively studied for its role in preventing PVR. It is an antineoplastic agent that inhibits enzymes necessary for deoxyribonucleic acid (DNA) synthesis and cellular proliferation. MTX has been routinely used for conditions like psoriasis, rheumatoid arthritis, keloids, juvenile idiopathic arthritis, sympathetic ophthalmitis, sarcoidosis-related panuveitis, mucous membrane pemphigoid, resistant uveitis, and primary intraocular lymphoma. MTX has the potential to block various aspects of PVR formation including fibrosis and cellular proliferation [3].

MTX has been recently found to inhibit PVR by stopping cellular proliferation and promoting organized apoptosis in an in vitro model. MTX has also been found to be effective in lowering the incidence of PVR when used as an adjunct in irrigation fluid during vitrectomy for retinal detachment [17]. MTX has been found safe in silicone oil and is being currently studied using consecutive intravitreal injections in silicone oil-filled eyes [3]. 

We have conducted a study where the use of MTX in the form of continuous infusion during surgery was evaluated in the prevention of recurrent RD and functional outcomes in high-risk patients of RD. Since PVR is known to start as early as two weeks with a median of two months, we have chosen to give only a single dose of MTX during surgery. 

## Materials and methods

This prospective interventional case series was conducted at Jinnah Hospital in Lahore, Pakistan from January 2021 to February 2021. This cohort consisted of 30 eyes of 30 patients who received MTX in irrigation fluid during vitrectomy for RD repair. All patient data were recorded on a predesigned proforma. PVR was graded according to The Retina Society Terminology Committee, 1991. The study was approved by the Ethical Review Board of Jinnah Hospital, Lahore (Ref # 19/07/01/2021/S2 ERB). A detailed and informed consent was sought from all patients where all alternatives, complications, and prognoses were explained. We collected relevant data of patients in strict compliance with the Declaration of Helsinki. 

**Figure 1:**
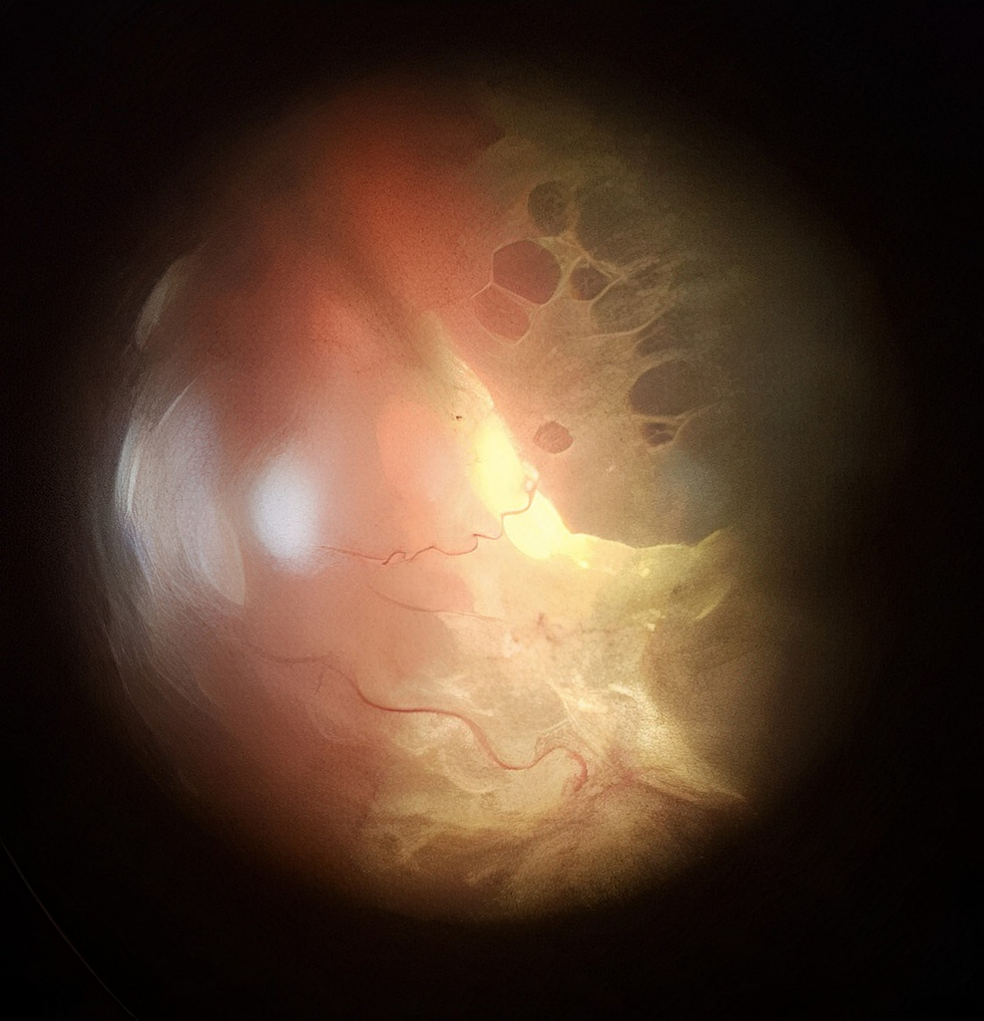
Posterior and diffuse Grade C PVR with full-thickness retinal folds

**Figure 2:**
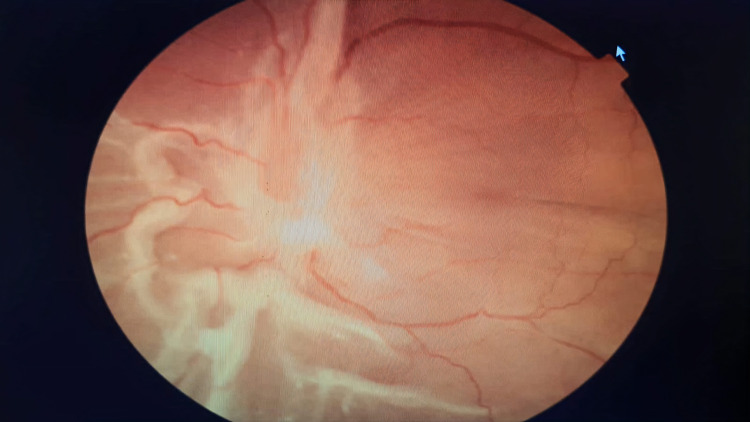
Posterior and diffuse Grade C PVR with full-thickness retinal folds and subretinal strands

All patients were operated on by a single surgeon (QLC). All patients were evaluated by two vitreoretinal surgeons (SJ and QLC). Only patients having Grade C PVR (as per The Retina Society Terminology Committee, 1991) in at least one quadrant were included in this study. Figures 1 and 2 show the type of cases included in this study.

We selected all patients through a non-purposive consecutive sampling technique and the following inclusion and exclusion criteria were used:

Inclusion criteria

One or more than one previous RD surgery and recurrent RD with Grade C PVR

Penetrating ocular trauma with RD

Retinal detachment with preoperative Grade C PVR

Exclusion Criteria

Faulty projection of light

Previous glaucoma surgery

History of adverse reaction to MTX

Severe dry eye or a corneal disease

Surgical technique

We used 80mg of MTX in 1000mL of balanced salt solution. This dose was selected to mimic the dose of MTX for the treatment of intraocular lymphoma (400ug of MTX in ~5 mL of the human eye) [18]. All surgeries were performed using a standard 23-gauge vitrectomy system (ACCURUS Surgical System; Alcon Forth Worth, TX, USA). The surgeon employed the use of retinectomies, perfluorocarbon liquid, scleral buckle, gas, and silicone oil as per the clinical needs. Since this study did not have a control group, the last two year's retinal reattachment of QLC was 69.7 % for the type of cases included in this cohort. 

The outcome measures included four-month postoperative visual acuity (logMAR) and rate of retinal reattachment. We used Statistical Package for Social Sciences (SPSS) Version 20.0 (IBM Corp., Armonk, NY, USA) statistical software for data analysis. The Student’s t-test was used for comparison of pre and postoperative data. A p-value of <0.05 was considered significant. 

## Results

**Figure 3:**
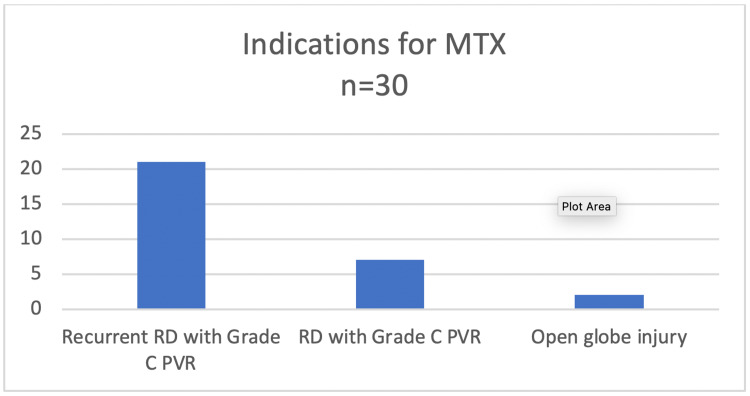
Indications for use of MTX infusion in advanced RD cases

This study included 30 eyes of 30 patients. The mean ± standard deviation age of the patients was 54.7 ± 10.2 years. Seventeen (56.66%) patients were females and 13 (43.34%) were males. The average number of previous retinal surgeries was 1.3 (range 0-4). The indications for use of MTX during PPV are summarized in Figure 3.

**Figure 4:**
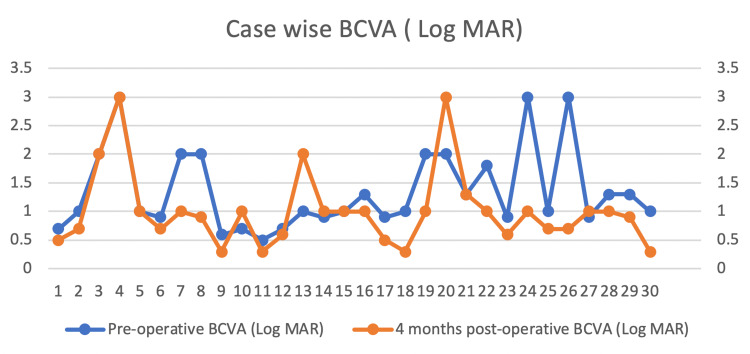
Comparison of preoperative and four months postoperative visual acuity in logMAR

All surgeries were performed using a 23-gauge vitrectomy system. Membrane peeling, endolaser, and silicone oil (1000Cs) were used in all eyes. Six (20%) eyes received an encircling scleral buckle as well. Thirteen eyes (43.33%) underwent simultaneous phacoemulsification and intraocular lens (IOL) implant at the time of vitrectomy. The rest of the patients had IOLs implanted earlier. Four (13.3%) eyes received relaxing retinotomies and retinectomies to achieve per-operative retinal flattening. The case-wise preoperative and postoperative best-corrected visual acuity (BCVA) at the four-month follow-up is summarized in Figure 4.

**Figure 5:**
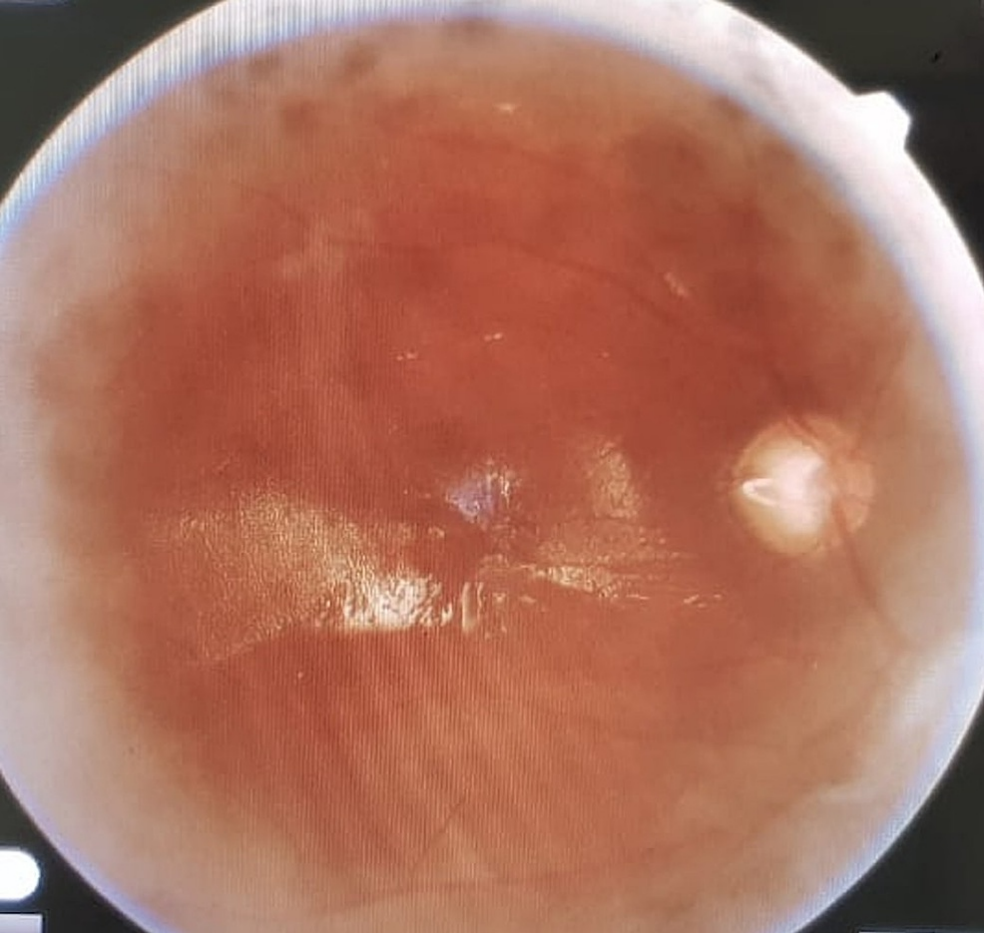
Postoperative retinal reattachment in a silicone oil-filled eye at the four-month follow-up

**Figure 6:**
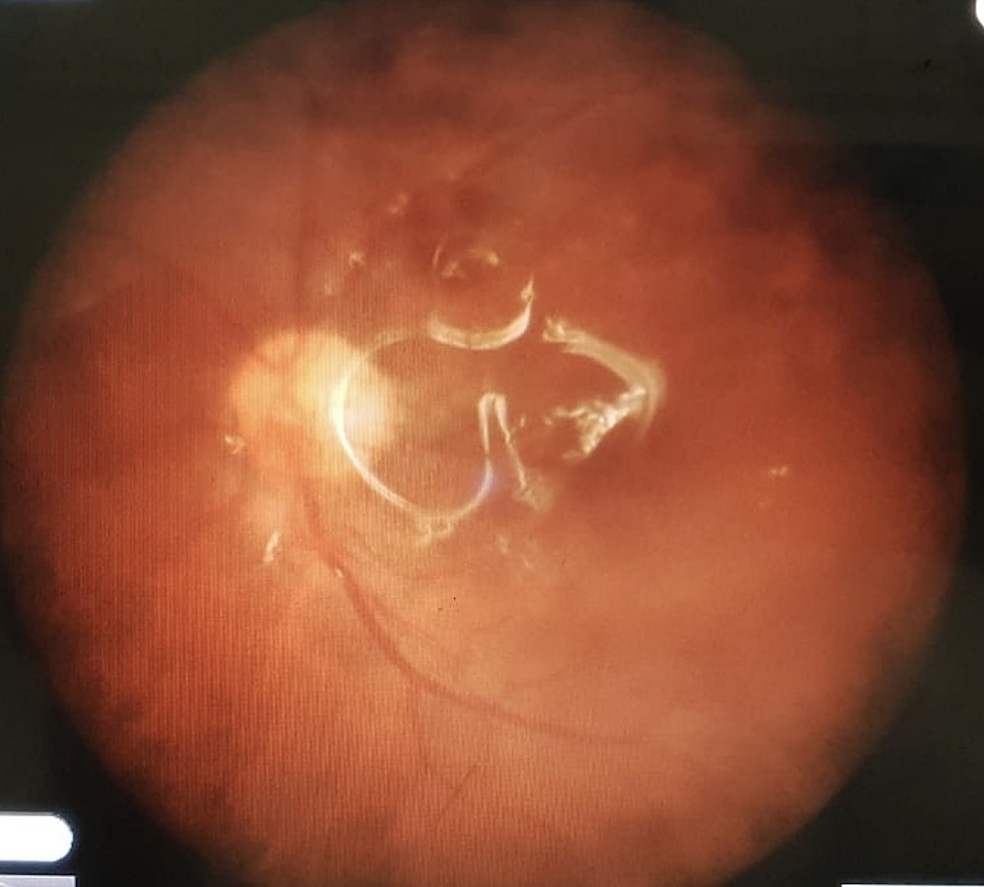
Postoperative retinal reattachment in a silicone oil-filled eye at the four-month follow-up

Postoperative retinal reattachment status at the end of follow-up is shown in Figures 5 and 6.

The mean preoperative BCVA was 1.35 logMAR (range 0.5-3). Mean four months postoperative BCVA was 1.01 logMAR (range 0.3-3) (Student’s t-test; p-value <0.05). Postoperative BCVA remained unchanged in five (16.6%) eyes, improved in 20 (66.6%), and deteriorated in five (16%) eyes. Three out of five eyes that had deterioration in postoperative BCVA were the ones that developed re-detachment after this surgery. A total of six (20%) patients developed retinal re-detachment at an average of 44.16 days after surgery. The remaining 24 (80%) retinas remained attached at the four-month follow-up. Four out of the six re-detached retinas had additional PVR that led to re-detachment. One eye had severe hypotony after surgery that led to retinal detachment and one eye had insufficient laser application identified as the cause of re-detachment. All eyes that developed re-detachment of retinas were operated on again. In the consecutive PPV, MTX was used again in infusion form as described earlier. Retinal reattachment was achieved in four eyes whereas one eye developed phthisis and one retina remained detached after repeat surgery. For these additional surgeries, we used a scleral buckle, additional endolaser, and retinectomies in an attempt to achieve retinal reattachment. All patients receiving relaxing retinotomies and retinectomies in the first surgery maintained retinal attachment after the follow-up. 

Seventeen (56.6%) eyes had pre-operative BCVA of 1.0 whereas 25 (83.3%) had BCVA 1.0 at four months postoperatively. Six (20%) patients had preoperative BCVA of 0.7 whereas 12 (40%) patients had BCVA 0.7 at four months postoperatively. 

Seven (23.3%) eyes had elevated intraocular pressure at one day and one week, post-operatively, and all these elevated pressures were controlled with topical antiglaucoma medications (Betoxolol 0.5% and Dorzolamide 2%). No patient developed any known complications related to the use of MTX. There were no cornea-related complications to be reported. At the end of the four-month follow-up, we observed that three (10.7%) of the attached retinas developed additional PVR that did not lead to recurrent retinal detachment. 

## Discussion

All patients who present with RD and have associated risk factors such as repeated ocular or retinal procedures, open globe trauma, preexisting PVR in an old RD, aphakia, heavy laser or cryotherapy burns, uveitis, hypotony, subretinal or vitreous hemorrhage, and choroidal detachment, are at higher risk of ensuing PVR [7,9]. For the simplicity of this study, we carefully chose only those patients who had Grade C PVR at presentation, had multiple failed retinal reattachment surgeries or had open globe injuries. This is in contrast to a similar study conducted by Sadaka et al. who included patients with risk factors beyond ours. They also included patients with tractional retinal detachment whereas our study only included patients with rhegmatogenous RD [3]. Both studies were single-arm investigations using MTX in the infusion line and comprise a limited number of patients. The infusion line-assisted MTX dosing was chosen to ensure a uniform amount of MTX in the vitreous cavity as opposed to uncertain concentrations when given as a bolus at the end of surgery in gas or silicone oil. Also, this ensured that there was no aqueous layer underneath the tamponade and around the breaks at the end of surgery. 

In our study, we achieved 80% structural success after one surgery and 93.3% success after the second surgery. These results are comparable to Sadaka et al. who reported 90% structural success after one surgery using MTX in infusion line. In our study, we reported an improvement in BCVA at four months in 20 (66.6%) eyes and a stable BCVA in five (16.6%) eyes. Sadaka et al. reported similar results with improved vision in 21 (72.4%) and stable vision in five (17.2) patients. Their group overall responded better to MTX infusion because of more diverse inclusion criteria that included patients with tractional retinal detachment and uveitis. But, this choice of patients can also limit the ability to conclude the effectiveness of MTX in preventing PVR due to varying presentations. As opposed to this, our study has a focused inclusion criterion which shows useful results in a limited variety of presenting pathology. Both studies reported no side effects related to intraocular use of MTX. 

In a similar study conducted by Sampas et al., their group reported no cornea-related toxicity or other side effects when MTX was injected at weekly intervals for eight weeks. Although their group did not report any significant change in BCVA pre and post-surgery that can be limited due to their very short follow-up period, which was eight weeks [19].

In another study by Falavarjani et al., they conducted a comparative analysis by having a control group. Their intervention was different from our study where they injected 250ug of MTX in silicone oil at the end of the surgery. Their group did not report a statistically significant difference in the PVR-associated detachment rates in experimental and control groups. This may have been limited due to the small sample size in both groups. But the study also did not report any adverse events due to the use of MTX in a bolus form. Although there was only one [4,5] case of detachment in the experimental group, they did not report a significant change in BCVA in the control and experimental group postoperatively (p-value = 0.15). This study has a better design than our study due to the presence of a control group and a longer period of follow-up. Also, they removed the silicone oil in five eyes in the MTX group and did not report re-detachment [20]. No MTX-associated side effects were reported. 

In a similar cohort of five patients, Benner et al. reported a 100% reattachment rate in patients receiving repeated postoperative MTX injections on a weekly or fortnightly basis for many months. All patients had attached retinas at the 11 to 27 months follow-up period. Four (80%) out of five patients had BCVA > 20/200 at the end of the follow-up period. Despite excellent results, there are certain differences in our and Benner’s study. They used perfluorocarbon (PFCL) liquid at the end of the surgery as a tamponade that remained in the eye for four to five weeks. They did not report any PFCL-associated ocular toxicity [21]. 

Similarly, studies conducted by Nourinia et al. have also shown very encouraging and safe results of using MTX in differing protocols for preventing postoperative PVR [22}. Nourinia et al. reported 81.8% structural success at a mean follow-up period of nine months in their study of 11 patients who received 250ug intravitreal MTX in silicone oil-filled eyes at the conclusion of surgery and then at three and six weeks postoperatively. Their group reported no significant MTX-related side effects and functional improvement was also statistically significant. The results of this study are comparable to our study [22].

This brief review of various recently conducted studies shows that MTX is safe and effective in preventing PVR in the post-operative period of complex retinal reattachment surgeries. Previous studies have mentioned corneal complications, endophthalmitis, vitreous hemorrhage, and maculopathy as rare side effects of MTX [3]. Regardless of the mode of MTX being used (infusion, bolus, serial postoperative injections), it has proven itself to be safe. 

Gain Understanding Against Retinal Detachment (GUARD) is a randomized controlled trial evaluating the effectiveness of MTX in preventing PVR in a larger and more controlled setting. It is utilizing serial postoperative injections of MTX for the treatment group and has included recurrent RD cases with Grade C PVR and cases of open globe injury. The MTX group will receive a total of 13 injections in 16 weeks. The results of this study will further clarify the safety and efficacy of MTX use for the prevention of PVR. 

Our low rate of recurrent RD after MTX use and statistically significant improvement in BCVA is comparable to other studies using similar pharmacological intervention to curtail PVR. Other studies have also effectively proven the efficacy of MTX in RD associated with uveitis which essentially expands the indications of intravitreal use of MTX. Although the strengths of our conclusions are limited due to the small sample size, short follow-up duration, lack of control group, and possible patient selection bias, we have demonstrated a successful structural and functional outcome that is comparable to other studies. The strengths include its prospective study design, a single operating surgeon with standard operating protocols, comparable surgical duration, and a relatively homogenous cohort of the sample group. 

## Conclusions

As PVR will remain at high risk for failure of primary RD surgery, off-label use of MTX shall be considered in selected cases to mitigate the chances of PVR formation. We believe that more rigorous trials accounting for the dose, methodology, grade, and severity of PVR and other confounding factors need to be carried out to further evaluate if MTX has a place in the armamentarium of a retina surgeon to combat their most feared enemy - PVR. We also anticipate encouraging results from the GUARD trial that will inform us further about the safety and suitability of MTX use in advanced cases of RD.
